# Acute Lymphoblastic Leukemia in Combined Methylmalonic Acidemia and Homocysteinemia (cblC Type): A Case Report and Literature Review

**DOI:** 10.3389/fgene.2022.856552

**Published:** 2022-04-14

**Authors:** Jun Zhu, Shuisen Wan, Xueqi Zhao, Binlu Zhu, Yuan Lv, Hongkun Jiang

**Affiliations:** ^1^ Department of Pediatrics, The First Hospital of China Medical University, Shenyang, China; ^2^ Department of Pediatrics, West China Second University Hospital, Chengdu, China; ^3^ Department of Gynecology and Obstetrics, Shengjing Hospital of China Medical University, Shenyang, China

**Keywords:** methylmalonic acidemia, acute lymphoblastic leukemia, congenital heart diseases, homocysteinemia, genetic analysis

## Abstract

**Background:** Methylmalonic acidemia (MMA) can display many clinical manifestations, among which acute lymphoblastic leukemia (ALL) has not been reported, and congenital heart disease (CHD) is also rare.

**Case presentation:** We report an MMA case with ALL and CHD in a 5.5-year-old girl. With developmental delay and local brain atrophy in MRI, she was diagnosed with cerebral palsy at 9 months old. Rehabilitation was performed since then. This time she was admitted to hospital because of weakness and widespread bleeding spots. ALL-L2 (pre-B-cell) was confirmed by bone marrow morphology and immunophenotyping. Echocardiography showed patent foramen ovale. The girl was treated with VDLD and CAML chemotherapy, during which she developed seizures, edema and renal insufficiency. Decrease of muscle strength was also found in physical examination. Screening for inherited metabolic disorders showed significantly elevated levels of methylmalonate-2, acetylcarnitine (C2), propionylcarnitine (C3), C3/C2 and homocysteine. Gene analysis revealed a compound heterozygous mutaion in *MMACHC* (NM_015,560): c.80A > G (p.Gln27Arg) and c.609G > A (p.Trp203*). CblC type MMA was diagnosed. Intramuscular injection of cyanocobalamin and intravenous L-carnitine treatment were applied. The edema vanished gradually, and chemotherapy of small dosage of vindesine was given intermittently when condition permitted. 2 months later, muscle strength of both lower limbs were significantly improved to nearly grade 5. The levels of methylmalonic acid and homocysteine were improved.

**Conclusion:** Metabolic disease screening and gene analysis are very necessary for diseases with complex clinical symptoms. ALL can be a rare manifestation for MMA.

**Synopsis:** We report a case of methylmalonic acidemia with acute lymphoblastic leukemia and congenital heart disease, which uncovered the importance of genetic testing and metabolic diseases screening in patients with multiple systemic organ involvement.

## Introduction

Methylmalonic acidemia (MMA) is an autosomal recessive disorder of methylmalonate and cobalamin (cbl; vitamin B12) metabolism first reported in 1967. Two main forms and different subtypes of the disease were identified based on enzymic and metabolic abnormalities: methylmalonyl-CoA mutase (MCM) defects, caused by mutation in *MUT* gene, including MUT^0^ and MUT^−^ type; and synthesis and dysfunction of adenosylcobalamin (AdoCbl) and methylcobalamin (MeCbl), associated with mutation in *MMAA*, *MMAB*, *MMCHC*, *MMADHC*, *LMBRD1*, *HCFC1*, and *ABCD4* gene, including cblA, cblB, cblC, cblD, cblF, cblX, and cblJ type (Zhou et al., 2018). According to clinical and biochemical characteristics, MMA can also be classified into two phenotypes: isolated MMA, including *cblA*, *cblB*, *cblH*, and *MUT* deficiencies; and combined MMA, where the levels of both methylmalonic acid and homocysteine elevate, such as *cblC*, *cblD*, and *cblF* deficiencies (Watkins and Rosenblatt, 2013).

Abnormal accumulation of toxic metabolites can cause multisystem damages, including kidney, heart, eye, skin and nervous system, as well as gastrointestinal system and immune system. Symptoms may occur before 1 year of age or in early infancy (early-onset MMA), and may also appear between the ages of 4 and 14, even in adulthood (late-onset MMA). Clinical presentations are relatively non-specific, such as lethargy, seizures, developmental delay, hypotonia, movement disorders, feeding difficulties and vomiting (Zhou et al., 2018). Hematological disorders have been reported in some cases, such as megaloblastic anemia, neutropenia or pancytopenia. Cardiovascular changes have also been observed in few cases, such as cardiomyopathy and arrhythmias.

In this study, we reported a combined MMA (cblC type) case in a 5.5-year-old girl, with rare complications -- acute lymphoblastic leukemia (ALL) and patent foramen ovale (PFO). To our knowledge, no such complication has ever been described.

## Case Presentation

The patient was a 5.5-year-old girl, whose primary symptom was developmental retardation—unable to crawl at 9 months. With no history of intrauterine infection or postnatal asphyxia, the girl was diagnosed with cerebral palsy by local hospital based on brain MRI changes. After receiving aggressive rehabilitation, she was barely able to walk without help until nearly 3 years old.

In January 2021, the girl was brought into hospital again because of weakness and widespread bleeding spots for 1 week. Pale lips and face, petechiae in skins and edema in eyelids were observed in physical examination. Blood routine examination showed severe anemia (hemoglobin of 53 g/L), moderate thrombocytopenia (platelets of 22×10^9^/L), with normal white blood cell count (6.53×10^9^/L). Liver function, kidney function and myocardial enzyme tests were all normal. Proteinuria (+) was detected, and 24-hour urine protein was 0.38 g. Echocardiography showed patent foramina ovale of 3.4 mm. In order to find out the reason of hematological abnormalities, bone marrow puncture was performed. Morphology and immunophenotyping confirmed the diagnosis of ALL-L2 (pre-B-cell). Ph-like ALL gene analysis showed positive mutated *IKZF1* gene, lower expression of *CRLF2* gene and positive mutated *WTI* gene. No abnormal karyotype was found in chromosome analysis. VDLD regimen (vindesine, daunorubicin, L-asparaginase and prednisone) was used as induction chemotherapy. Complete remission (CR) was achieved 15 days later, with the mutated *IKZF1* gene turning negative.

**FIGURE 1:**
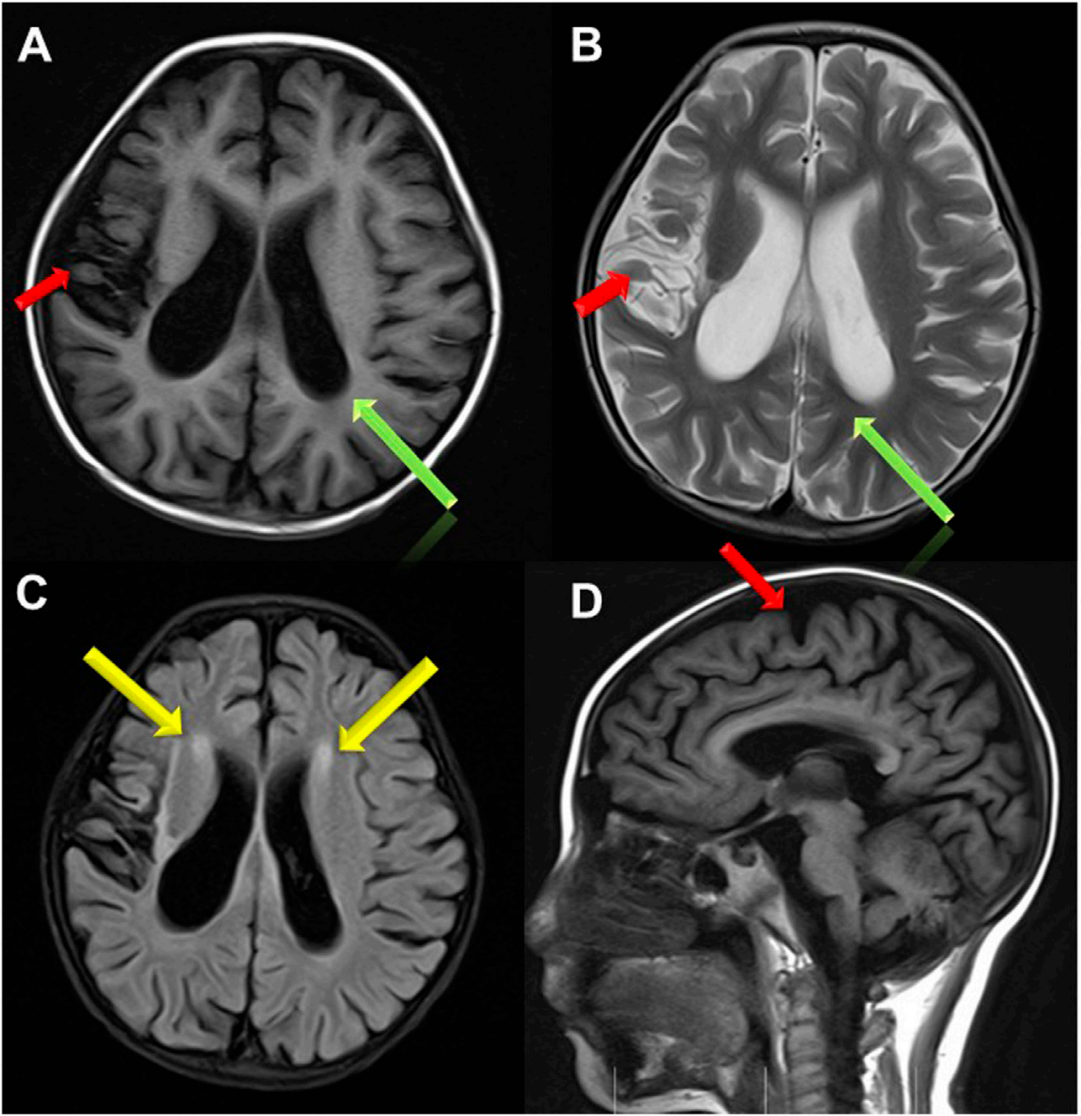
Brain MRI images. **(A,B,D)** Brain atrophy was found in Transverse T1 and T2 weighted image and Sagittal T1 weighted image(the red arrow); Sulci fissure widened and deepened in Transverse T1 and T2 weighted image (the green arrow); **(C)** High signal in white matter near to bilateral periventricular in FLAIR sequence (the yellow arrow).

Treatment for leukemia continued for 2 months, when the girl developed vomiting, abdominal pain, distension and muscle tension during the early intensive CAML chemotherapy (cyclophosphamide, cytarabine, mercaptopurine and pegaspargase). Severe acute pancreatitis was diagnosed based on elevated serum amylase, lipase and characteristic abdominal CT changes. At same time, she showed recurrent attack of generalized seizures. Ambulatory electroencephalogram revealed lateralized periodic epileptic discharges in the left brain, and partial discharges from the left back side. Brain magnetic resonance imaging (MRI) showed encephalomalacia and gliosis in the left frontal and parietal lobe and basal ganglia, with regional brain atrophy, and abnormal signals in the left lateral ventricle and semicovale region (Figure 1). She also showed edema, hypertension, increased urinary protein (+++, 2.035 g/24 h) and hematuria (blood test +++, RBCs 25.52/HP). Chemotherapy was halted to deal with pancreatitis, seizures and renal injury. After her condition improved, the girl was discharged with prescriptions of oral levetiracetam, nifedipine, prednisone and furosemide.

1 month later, she showed gradually aggravated edema, hypertension, renal dysfunction, oliguria (about 200ml/24 h) and three episodes of seizures. Physical examination results at this admission were: BP 133/102 mmHg, severe edema in both eyelids and lower limbs, pale lips and face, and decrease of muscle strength (grade 3 in upper limbs, grade 2 in lower limbs), with normal muscle tone. Massive proteinuria (++++, 2.255 g/24 h) and hematuria (blood test +++, RBCs 38.42/HPF) was detected. Peripheral blood test results were: WBC of 8.26×10^9^/L (4-10×10^9^/L), Neutrophil of 6.24×10^9^/L (1.8-6.3×10^9^/L), hemoglobin of 85 g/L (110–140 g/L), platelets of 65×10^9^/L (100-300×10^9^/L), albumin of 28.9 g/L (40–55 g/L), lactic dehydrogenase (LDH) of 2963U/L (120–250U/L), urea of 14.36 mmol/L (2.85–7.14 mmol/L) and creatinine of 88 μmol/L (59–104 μmol/L). Calculated creatinine clearance (Ccr) was 36 ml/min. Based on these results, the girl was diagnosed with CKD(G3b).

**FIGURE 2:**
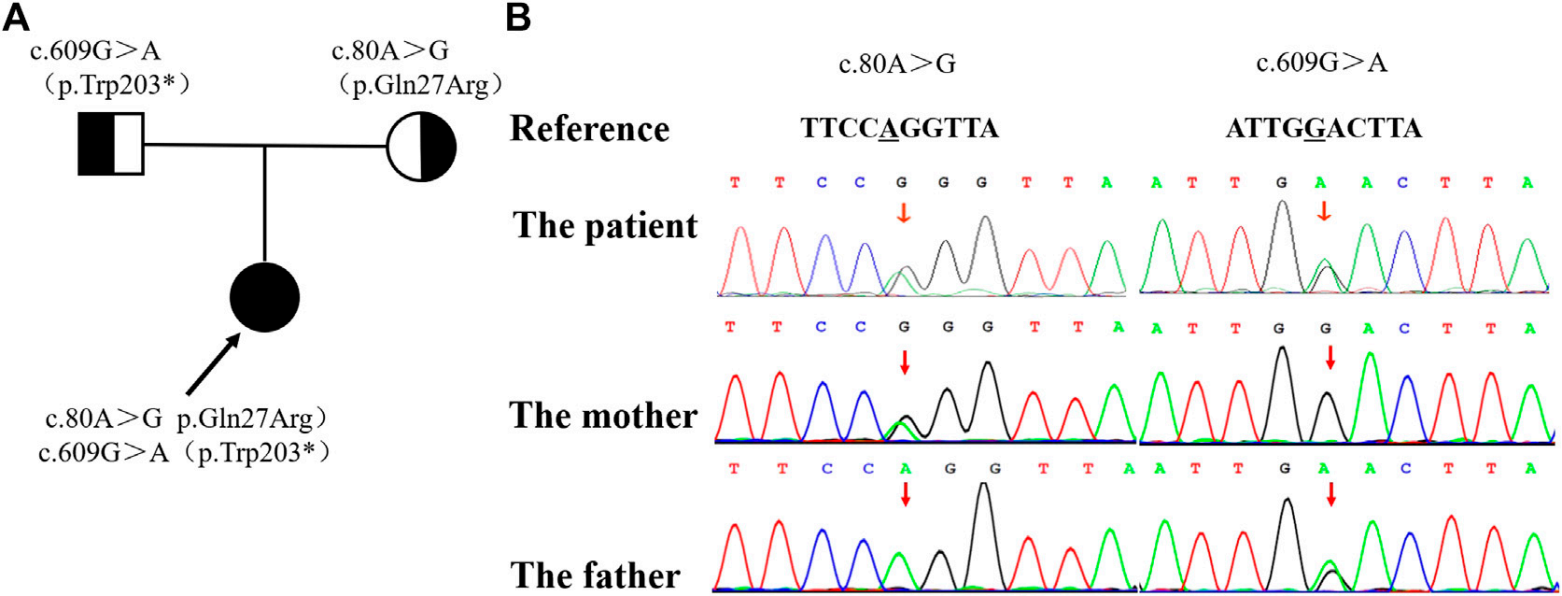
The family pedigree showing the mutations detected in *MMACHC*. **(A)** The pedigree of the family with MMA. The arrow indicates the proband; her parents have no signs of MMA. **(B)** The mutations detected in the family. The proband has both mutations, while the c. 80A >G mutation was only detected in her mother and the c. 609G > A mutation was only detected in her father.

Considering the early-onset of symptoms and multisystem involvement, screening for metabolic diseases was performed. Results are: methylmalonic acid-2 of 8.68 umol/L (0.2-3.6 umol/L), serum homocysteine of >50 umol/L (4.44-13.56 umol/L), free carnitine of 177.194 umol/L (10-60 umol/L), acetylcarnitine (C2) of 58.959 umol/L (6-30 umol/L), propionylcarnitine (C3) of 17.561 umol/L (0.5-4 umol/L), and C3/C2 of 0.298 (0.04-0.25). MMA was suspected, whole exome sequencing (WES) was performed, and detected mutations was verified by Sanger sequencing. The proband had a compound heterozygous mutation in MMACHC gene, c.80A > G (p.Gln27Arg) in exon 1 inherited from her mother, and c.609G > A (p.Trp203*) in exon 4 [reference sequence: NM_015560) from father (Figure 2). c.609G > A(p.Trp203*]is a nonsense mutation, which is expected to change the 203rd amino acid of encoded protein from Trp to stop codon, causing the encoded protein to be truncated and lose its normal function. As was reported in several literatures, this mutation is a hot mutation in Chinese patients with methylmalonic acidemia and homocysteinemia. In the gnomAD database, the frequency of this variation in East Asian population is 0.0005562. c. 80A > G (p.gln27arg) is a missense mutation, which is expected to change the 27th amino acid of the encoded protein from Gln to ARG. The frequency of this mutation in gnomAD database is 0.0001669 in East Asian population. Considering the ACMG score, both variants are pathogenic.

The final diagnosis was determined as combined methylmalonic acidemia and homocysteinemia (cblC type), secondary nephrotic syndrome (nephritis type), secondary hypertension (level 2), and chronic kidney disease (C3b). Vitamin B12 (cyanocobalamin; 1mg, twice-weekly) and L-carnitine (1000 mg, IV, qd) were given, together with oral antiepileptic drug, antihypotensive agents, diuretics and prednisone. When condition permitted, a low dose of vindesine (1.5 mg, IV) was administered intermittently for chemotherapy. About 1 week later, the edema vanished gradually. After 2 months of treatment of vitamin B12 and L-carnitine, the muscle strength of both lower limbs were significantly improved to nearly grade 5. In the following 3 months, the girl was treated with Chimeric Antigen Receptor T-Cell Immunotherapy (CAR-T) twice. After reinfusion of her own T cells, there was no abnormality in bone penetration, MRD and fusion gene. Then she received sequential treatment of CD19 and CD22, and 3 times of VDL chemotherapy. Now the girl receives intramuscular injection of cyanocobalamine twice a week, oral administration of L-carnitine and folic acid tablets. The levels of methylmalonic acid and homocysteine were improved.

## Discussion

Methylmalonic acid is the metabolite of methylmalonyl-CoA in the catabolism of branched chain amino acids (isoleucine, methionine, threonine and valine), odd-chain fatty acids, gut-derived propionate and cholesterol. In order to enter the tricarboxylic acid cycle, methylmalonyl-CoA is reversibly isomerised to succinyl-CoA, which process is catalyzed by mitochondrial enzyme methylmalonyl-CoA mutase and requires adenosylcobalamin (AdoCbl) as an essential cofactor (Froese et al., 2015; Hu et al., 2018). Children with cblC type MMA have defects in *MMACHC* gene, whose coding proteins is related with the synthesis of Adocbl and methylcobalamin (MeCbl) (Bassila et al., 2017). MeCbl is the coenzyme of methyltetrahydrofolate-homocysteine methyltransferase, also known as methionine synthase (MS), located in the cytoplasm, catalyzing the methylation of homocysteine to methionine. Low levels of AdoCbl and MeCbl in cblC type patients cause abnormal accumulation of metabolites such as methylmalonate, methylcitrate and 3-hydroxybutyric acid, decreased serum methionine, and reduced activity of succinic dehydrogenase. As a result, mitochondrial energy metabolism was affected, and multiple symptoms appear (Bassila et al., 2017).

In China, most of *MMACHC* mutations are located in exons 3 and 4, and C. 609G > A (P.w203x) is the most common. In this case, clinical manifestations include cerebral palsy, developmental delay, epilepsy, hypertension, proteinuria, chronic renal insufficiency; laboratory findings showed elevated methylmalonic acid-2, acetyl carnitine, propionyl carnitine in blood and urine; genetic testing revealed a compound heterozygous mutation in *MMACHC* gene [c. 80A > G (P.q27r, from her mother) and c. 609G > A (P.w203x, from her father)], which has been reported in MMA cases before (Hu et al., 2018; Wang et al., 2019). Clinical features, laboratory findings and genetic testing all support the diagnosis of cblC-type MMA.

The diagnosis of MMA mainly depends on examination of blood acylcarnitine profile and urinary organic acids, characterized by remarkably increased blood C3, C3/C2 ratio and relatively high concentrations of methylmalonic acid and methyl citrate in urine (Keyfi et al., 2016). Genetic analysis is helpful for the classification of MMA (Chandler and Venditti, 2005). According to responses to vitamin B12, MMA can be divided into two sub-type: Vitamin B12 responsive and vitamin B12 unresponsive. B12 responsive ones are mostly caused by deficiency of coenzyme synthesis (Froese et al., 2009), while unresponsive ones are mostly caused by deficiency of mutase (Pela et al., 2006). Once MMA was suspected or diagnosed, initial treatments must be performed without delay. Basic principle of treatment is to reduce the production of methylmalonic acid and its bypass metabolites, and to accelerate their clearance. For acute management, treatments are stabilizing the patient, restricting protein intake, providing enough calories, and administering drugs including L-carnitine, vitamin B12 (preferably hydroxo-Cbl), biotin, sodium phenylbutyrate and arginine. For long-term management, vitamin B12 responsive MMA patients should be treated with vitamin B12 (1mg, IM, once or twice a week), followed by other oral drugs including vitamin B6, betaine, folic acid, L-carnitine, and sometimes methionine. If concentration of C3 and urinary methylmalonic acid are at appropriate levels, highly restricted diet may not necessarily be taken (Ribas et al., 2010). For vitamin B12 unresponsive cases, specialized amino acid formulations (containing minimal to no isoleucine, methionine, threonine, and proline, etc.) should be used, and nutritional intervention, such as strict restriction on the intake of natural protein and supplements, should be applied. L-carnitine is effective for both types of MMA because of its ability of promoting the excretion of both methylmalonic acid and C3 (Ribas et al., 2010). After diagnosis, the case was treated with intramuscular injection of vitamin B12 (1mg, twice a week), supplemented by L-carnitine (1g/day). The clinical course should be classified as early-onset: developmental delays in gross movement, brain atrophy in cerebral MR images, and seizures. The results of metabolic screening and genetic analysis confirmed the diagnosis. Symptoms improved significantly after vitamin B12 administration, so we speculate this case to be an early-onset and vitamin B12 responsive one.

B-acute lymphoblastic leukemia (B-ALL) is the most prevalent childhood hematological malignancy, whose development is a complex process of multiple gene mutations, involving fusion mutations in early-stage and collaborative genes mutations in late-stage. In particular, gene mutations associated with B cell development play an increasingly important role in the pathogenesis of B-ALL (Pui et al., 2015).

We conducted a literature review about congenital hematological disease and cardiac disease in MMA. We searched for keywords “Hematological”, “Cardiovascular”, “heart”, “blood” and “child” in all fields in PubMed and Webofscience, and screening out original articles focusing on cardiovascular disease and hematological disease in MMA. This section was summarized in Tables 1, 2.

**TABLE 1:** Multi-case analysis of MMA with cardiovascular and hematological disease.

NO.	References	Year	MMA type	Population origin	Total number	Hematological system performance	Cardiovascular system performance	Treatment	Outcome
1	Chao Wang	2021	NM	China	75	Anemia 23 (30.7%)	Cardiomyopathy 1 (1.3%)	NM	NM
						Pancytopenia 3 (4.0%),	PAH 1 (1.3%)		
						Granulocytopenia 15 (20%),	ASD 6 (8%)		
						Thrombopenia 6 (8%),	VSD 1 (1.3%)		
						Haemolytic uremic 2 (2.7%)	PDA 5 (6.7%)		
2	Ruxuan He	2020	NM	China	68	Anemia 27 (39.7%)	Cardiomyopathy 2 (2.9%)	CBL, CAR, BET	Lagerly Return
3	Lulu Kang	2020	NM	China	224	Anemia 66 (29.5%), Pancytopenia10 (4.5%)	Cardiomyopathy6 (2.7%), PAH 2 (0.9%)	CBL, CAR, PR	Lagerly return
4	Ruxuan He	2020	cblC	China	132	Anemia 37 (28%)	NM	CBL, CAR, BET	Lagerly return
5	Chao Wang	2019	cblC	China	28	Anemia 6 (21.4%), Granulocytopenia 3 (10.7%)	Atrial septal defect 3 (10.7%)	NM	NM
6	Yi Liu	2018	NM	China	1003	Anemia and haemolytic uremic260 (26.6%)	PAH17 (1.7%)	CBL, CAR, BET, PR	Lagerly return
7	Sabine Fischer	2014	cblC	Italy	86	Anemia 45 (53.3%), macrocytosis8 (7.9%), haemolytic uremic 4 (4.5%)	NM	BET, CAR, F	Lagerly return
				Portugal		Macrocytosis 18 (20.9%)			
				Spain		Haemolytic uremic 8 (9.3%)			
8	Fei Wang	2010	cblC	China	43	Anemia 36 (83.7%)	NM	NM	NM
9	Celia Nogueira	2008	cblC	Italy, Portugal	41	Anemia, thrombopenia and granulocytopenia 21 (51%)	NM	NM	NM

Abbreviation: PAH, pulmonary arterial hypertension; ASD, atrial septal defect; VSD, ventricular septal defect; PDA, patent ductus arteriosus; NM, not mentioned; CBL, hydroxocobalamin; CAR, carnitine; PR, protein restriction. BET, betaine. F, folic acid.

**TABLE 2:** Case reports of MMA complicated with cardiovascular diseases.

NO.	References	Year	Sex	At diagnosis age	MMA-type	MMACHC mutation	Types of heart disease	Mean Hey (umol/L)	Mean urine MMA (mmol/mol cr)	Treatment	Outcome
1	Ling-yi Wen	2020	female	12 years	NM	c.80A > G/c.609G > A	PAH and dilated right ventricle	155.8	↑	CBL, F, CYS	Largely return
2	Ya-Nan Zhang	2019	female	7 months	NM	NM	Dilated left ventricle	NM	NM	CBL, CYS	Largely return
3	Ya-Nan Zhang	2019	male	6 years	NM	NM	PAH and dilated right ventricle	NM	NM	CBL	died
4	Ya-Nan Zhang	2019	female	6 years	NM	NM	PAH and dilated right ventricle	NM	NM	CBL, CYS	died
5	Luciano De Simone	2018	male	2 years	cblC	c.271-272dupA/c.347 T > C	PAH and dilated right ventricle	74	↑	CBL, F, CYS, CAR	Largely return
6	Jun KIDO	2017	female	39 years	NM	NM	PAH and dilated right ventricle	NM	NM	NM	Recovery (4Y)
7	Jinrong Liu	2017	female	21 months	cblC	c.80A >G/c.331C > T	PAH and dilated right ventricle	>50	↑	CBL. CYS, F	Recovery (3Y)
8	Carlos E. Prada	2011	female	22 years	cblB	NM	Hypertrophic cardiomyopathy	NM	NM	NM	died
9	Carlos E. Prada	2011	male	2 years	Mut-0	NM	Dilated cardiomyopathy	NM	NM	CAR	died
10	Carlos E. Prada	2011	female	4 years	Mut-0	NM	Dilated cardiomyopathy	NM	NM	NM	died
11	Laurie E. Profitlich	2009	male	2 months	cblC	271dupA/271dupA	Mitral valve prolapse and Mild mitral regurgitation	95	266	CBL, PR, CYS, F	Largely return
12	Laurie E. Profitlich	2009	male	Prenatal	cblC	271dupA/271dupA	Focal left ventricular	107	196	CBL, PR, CYS, F	Largely return
13	Laurie E. Profitlich	2009	male	Birth	cblC	271dupA/271dupA	Normal structure	32	35	CBL, PR, CYS, F	Largely return
14	Laurie E. Profitlich	2009	female	Birth	cblC	271dupA/271dupA	Normal structure	30	34	CBL, PR, CAR, F	Largely return
15	Laurie E. Profitlich	2009	male	Birth	cblC	568insT/568insT	Secundum atrial septal defect	63	29	CBL, PR, CAR, F	Largely return
16	Laurie E. Profitlich	2009	female	2 months	cblC	G609A/G609A	Normal structure	35	24	CBL, PR, CYS, CAR, F	Largely return
17	Laurie E. Profitlich	2009	male	Birth	cblC	547-8delGT/285dupA	Secundum atrial septal defect	64	31	CBL, PR, CYS, CAR, F	Largely return
18	Laurie E. Profitlich	2009	male	Birth	cblC	G608A/G608A	Normal structure	42	20	CBL, PR, CAR, F	Largely return
19	Laurie E. Profitlich	2009	female	3 years	cblC	C666A/C666A	Normal structure	99	57	CBL, PR, CYS, ASA	Largely return
20	Laurie E. Profitlich	2009	male	3 months	cblC	C481T/C481T	Muscular ventricular septal defect	69	74	CBL, PR, CYS, ASA	Largely return
21	Isabelle De Bie	2009	female	27 years	cbIC	NM	VSD and dilated cardiomyopathy	236	↑	CBL, PR, CAR, F, CYS	Largely return
22	M. Tomaske	2009	female	2 weeks	cbIC	NM	VSD	282	1914	CBL, CAR, F, CYS	Largely return
23	Markus K. Heinemann	2001	female	19 days	NM	NM	VSD	NM	↑	CBL and Surgery	Largely return
24	Hans C. Andersson	1999	NM	5 year	cbIC	NM	Pulmonic stenosis	↑	↑	CBL	Largely return
25	Hans C. Andersson	1999	NM	2 years	cbIC	NM	VSD	↑	↑	CBL	Largely return

Abbreviation: PAH, pulmonary arterial hypertension; ASD, atrial septal defect; VSD, ventricular septal defect; PDA, patent ductus arteriosus; NM, not mentioned CBL, hydroxocobalamin; CAR, carnitine; PR, protein restriction. ↑, larger than the reference value. CBL, hydroxocobalamin; PR, protein restriction; CYS, cystadane; ASA, aspirin; CAR, carnitine; F, folic acid.

In summary, (Nogueira et al., 2008; Wang et al., 2010; Fischer et al., 2014; Liu et al., 2018; Wang et al., 2019; He et al., 2020a; He et al., 2020b; Kang et al., 2020; Wang et al., 2021), anemia is the most common blood system damage of MMA(Table 1), the incidence of which is 21.4–83.3%. Other hematological complications include granulocytopenia, thrombocytopenia, pancytopenia and hemolytic uremia.

The protein encoded by *MMACHC* is mainly involved in the biological synthesis of Adocbl and Mecbl (Bassila et al., 2017), and then affects the levels of methylmalonyl-coa mutase (MUT) and MMA, and also affects the activity of methionine synthase (MTR). MTR C. 2756 mutation has been reported to be positively correlated with the occurrence of ALL in Asian population in former studies (Yu et al., 2010), but its mechanism is still unclear.

Among the 14 methionine-dependent tumor lines tested by Watkins (Watkins, 1998), reduced methionine synthase function and reduced MeCbl and AdoCbl synthesis was observed only in MeWo LC1 melanoma cell lines. It was later proved that the decreased expression of MMACHC in MeWo LC1 was caused by methylation of CpG island at the end of the corresponding gene 5. Recent studies have shown that in some cblC patients, specific mutations in *PRDX1*, a contiguous gene of *MMACHC*, lead to secondary epigenetic mutations that affect methylation of the MMACHC promoter and expression of MMACHC (Guéant et al., 20172018). Both MeWo LC1 human melanoma cell line and cblC patient-derived cells have reduced AdoCbl and MeCbl synthesis, decreased intracellular cobalamin concentration, and impaired activity of these two cobalamine-dependent enzymes (Loewy et al., 2009). These similarities open up the possibilities to clarify whether there is a common mechanism of methionine dependence in cblC patient-derived cells and MeWo LC1 cell lines (Loewy et al., 2009).

The child suffered from methylmalonic acidemia, which leads to decreased expression of *MMACHC* gene, decreased intracellular cobalamin concentration, decreased activities of Adobe Cbl and MeCbl, two cobalamine-dependent enzymes, and increased levels of methionine and homocysteine, resulting in changes in blood microenvironment. Many syndromes can have complications of leukemia, of which the possible reason may be microenvironment changes caused by chromosomal, genetic and metabolic abnormalities. Simultaneous MMA and leukemia may be associated with abnormal amino acid levels and changes in blood microenvironment, or may be related to the genetic variation of MMACHC and MTR C. 2756 and the subsequent decrease of MTR activity. Further studies are needed to determine whether these changes lead to acute lymphoblastic leukemia. While, perhaps the presence of both ALL and cblC methylmalonic acidemia in this case may also be a coincidence.

In MMA cases combined with heart disease (Table 1), (Andersson et al., 1999; Heinemann et al., 2001; Tomaske et al., 2001; De Bie et al., 2009; Profitlich et al., 2009), cardiomyopathy(1.2–2.9%) and pulmonary hypertension (0.9–1.7%) were more common (Andersson et al., 1999; Heinemann et al., 2001; Tomaske et al., 2001; De Bie et al., 2009; Profitlich et al., 2009; Prada et al., 2011; Kido et al., 2017; Liu et al., 2017; De Simone et al., 2018; Wen et al., 2020; Zhang et al., 2020). While mitral valve prolapse, mild mitral regurgitation, focal left ventricular uncompacting, atrial septal defect, ventricular septal defect, patent ductus arteriosus and pulmonary artery stenosis were also reported (Table 2).

A study suggested that MTR polymorphisms (rs1770449 and rs1050993) may be associated with the risk of CHDs and modified the relation between maternal folate intake and CHDs (Deng et al., 2019). Three independent case-control studies in a total of 2,340 patients with CHD and 2,270 controls suggested that two regulatory variants of MTR, 2186T.G and +905G.A were associated with increased risk of CHD (Zhao et al., 2014). Patent foramen ovale, a secondary atrial septal defect, was detected in this case by echocardiography. We speculate that the main cause of this patent foramen ovale was MMA combined with homocysteine. The structural defects of heart in cblC MMA patients may be related to the increase of metabolites such as methionine, s-adenosine methionine and s-adenosine homocysteine, or the lack of methionine synthase.

This case was unable to crawl at 9 months old, and was diagnosed with cerebral palsy based on brain atrophy in brain MRI. Gestational age and birth weight were normal, and no history of postnatal asphyxia, hypoxia, hyperbilirubin encephalopathy, and intrauterine infections were found. Seizures occurred during chemotherapy, and the results of EEG suggested epilepsy. Therefore, when a patient showed cerebral palsy and epilepsy with no common causes, screening for metabolic diseases should be performed to exclude metabolic diseases.

## Conclusion

1. Metabolic screening and genetic analysis are necessary for cases with multiple system involvement.
2. Cerebral palsy may have multiple causes, and genetic metabolic diseases should be taken into consideration apart from common causes such as preterm birth, low weight, history of postnatal asphyxia, and hypoxia, neonatal hyperbilirubin encephalopathy and intrauterine infection.
3. MMA and homocysteinemia should be considered in the differential diagnosis of congenital cardiac defects. Routine and regular cardiovascular assessments should be performed in patients with MMA to identify cases with cardiac structural defect and cases with risks of thromboembolism and stroke.
4. The lack of specific clinical manifestations causes difficulties in MMA diagnosis. Physicians, especially pediatricians in high-prevalence areas, should raise awareness for the diseases, screen high-risk patients timely and perform blood carnitines and urine organic acids analysis, so as to make early diagnosis and treatments, and improve prognosis of the disease.

## Data Availability

The datasets for this article are not publicly available due to concerns regarding participant/patient anonymity. Requests to access the datasets should be directed to the corresponding author.
